# Magnetic Resonance Elastography and Computational Modeling Identify Heterogeneous Lung Biomechanical Properties during Cystic Fibrosis

**DOI:** 10.21203/rs.3.rs-4125891/v1

**Published:** 2024-03-21

**Authors:** Youjin Cho, Faisal Fakhouri, Megan N. Ballinger, Joshua A. Englert, Don Hayes, Arunark Kolipaka, Samir N. Ghadiali

**Affiliations:** The Ohio State University; King Saud University; The Ohio State University Wexner Medical Center; The Ohio State University Wexner Medical Center; Cincinnati Children’s Hospital Medical Center, University of Cincinnati College of Medicine; The Ohio State University Wexner Medical Center; The Ohio State University

**Keywords:** Lung Mechanics, Respiratory Disease, Finite Element Modeling, Heterogeneity, Strain Gradients, Shear Stiffness

## Abstract

The lung is a dynamic mechanical organ and several pulmonary disorders are characterized by heterogeneous changes in the lung’s local mechanical properties (i.e. stiffness). These alterations lead to abnormal lung tissue deformation (i.e. strain) which have been shown to promote disease progression. Although heterogenous mechanical properties may be important biomarkers of disease, there is currently no non-invasive way to measure these properties for clinical diagnostic purposes. In this study, we use a magnetic resonance elastography technique to measure heterogenous distributions of the lung’s shear stiffness in healthy adults and in people with Cystic Fibrosis. Additionally, computational finite element models which directly incorporate the measured heterogenous mechanical properties were developed to assess the effects on lung tissue deformation. Results indicate that consolidated lung regions in people with Cystic Fibrosis exhibited increased shear stiffness and reduced spatial heterogeneity compared to surrounding non-consolidated regions. Accounting for heterogenous lung stiffness in healthy adults did not change the globally averaged strain magnitude obtained in computational models. However, computational models that used heterogenous stiffness measurements predicted significantly more variability in local strain and higher spatial strain gradients. Finally, computational models predicted lower strain variability and spatial strain gradients in consolidated lung regions compared to non-consolidated regions. These results indicate that spatial variability in shear stiffness alters local strain and strain gradient magnitudes in people with Cystic Fibrosis. This imaged-based modeling technique therefore represents a clinically viable way to non-invasively assess lung mechanics during both health and disease.

## Introduction

The lung is a dynamically active organ and under normal physiological conditions, contraction of diaphragmatic muscles during inspiration and chest wall relaxation during expiration results in cyclic inflation/deflation and deformation of lung tissue(Levitzky 2018). In healthy subjects, movement of lung tissue during breathing exposes cells within the lung microenvironment to cyclic tensile strains (i.e. stretching deformations). Normal physiological strains (~ 5–10% magnitude) promote homeostatic cellular responses including surfactant production, epithelial cell proliferation and differentiation and prostacyclin production(Liu et al. 1993; Diem et al. 2020; Sanchez-Esteban et al. 2001; Skinner, Somervell, and Olson 1992). However, the degree of tissue deformation and tensile strain is highly dependent on the local stiffness of lung tissue. During chronic lung disease altered lung tissue stiffness may significantly alter the degree of tensile strain. As a result, developing non-invasive tools that can monitor changes in the lung’s biomechanical properties, including both stiffness and strain, during disease progression may lead to innovative ways to diagnose and/or predict the progression of lung disease.

It is well established that several respiratory diseases, e.g. fibrosis/cystic fibrosis, chronic obstructive pulmonary disease (COPD) and lung cancer, involve changes in lung tissue stiffness(Nho et al. 2022; Colasanti et al. 2004; Shukla et al. 2016) and non-invasive assessment of global lung stiffness is a major area of clinical research. Magnetic resonance elastography (MRE) is a noninvasive imaging method that uses phase-contract (PC) magnetic resonance imaging (MRI) to measure shear stiffness in various organs such as lungs, pancreas, liver, and breast (Fakhouri, Dong, and Kolipaka 2019; Mariappan et al. 2011; Kolipaka et al. 2017; Huwart et al. 2006; Balleyguier et al. 2018). MRE has been used to quantify lung stiffness in large animal models(Fakhouri et al. 2023) and human subjects(Gandhi et al. 2019; Kolipaka et al. 2017) documenting higher mean lung stiffness at total lung capacity (TLC) compared to residual volume (RV)(Fakhouri, Dong, and Kolipaka 2019). Unlike previous studies which used inverse techniques to estimate lung stiffness from CT-based measurements of lung deformation(Hasse et al. 2020; Hasse et al. 2018), MRE is a direct measure of lung tissue stiffness and can also resolve spatial and temporal variations in lung stiffness.

In addition to lung tissue stiffness, changes in both tensile strain magnitude and spatial strain gradients during respiration have emerged as important biomarkers of disease. Specifically, altered tensile strain during disease has been shown to alter several mechanobiological processes including epithelial/endothelial cell injury, activation of pro-inflammatory pathways and cancer cell invasion(Gattinoni et al. 2017; Waters, Roan, and Navajas 2012; Kelly et al. 2019; Lopez-Alonso et al. 2021; Higuita-Castro et al. 2014; Higuita-Castro et al. 2016). Furthermore, most pulmonary diseases involve heterogenous changes in lung stiffness(Hurtado et al. 2020; Hurtado et al. 2017) and this may lead to both heterogenous distributions of strain and large spatial gradients in strain within the deforming lung. Since spatial strain gradients have been shown to alter fibroblast migration, orientation and contractility(Chagnon-Lessard et al. 2017; Hsieh et al. 2014; Yang et al. 2023), advanced diagnostic tools that can non-invasively monitor heterogenous distributions of strain and strain gradients may provide additional information about disease progression.

Although previous investigators have used computational and finite element models to estimate lung deformation and tensile strain(Eom et al. 2010; Tehrani et al. 2015; Villard et al. 2005; Werner et al. 2009; Yang et al. 2019), these models assume homogenous stiffness distributions(Tehrani et al. 2015; Eom et al. 2010; Yang et al. 2019) and cannot capture the spatial variations in lung deformation observed *invivo*(Hurtado et al. 2020) and therefore cannot be used to quantify spatial variations in strain or strain gradients. In this study, we developed a novel technique that uses MRE and Finite Element Modeling (FEM) to non-invasively assess regional lung stiffness (i.e. shear modulus), strain magnitude, and spatial strain gradients and then apply this technique to assess lung biomechanical properties in healthy adults and in people with Cystic Fibrosis (pwCF). We first used MRE to non-invasively measure the heterogenous distributions of lung tissue stiffness and then integrate these stiffness measurements into a novel FEM model of lung deformation during the respiratory cycle. We hypothesize that accounting for heterogeneous spatial stiffness patterns will lead to higher strain gradients and that computational models which use homogenous properties will underestimate these strain gradients. We also hypothesize that pwCF will exhibit higher stiffness/shear moduli, reduced strain magnitude and reduced strain gradients within the consolidated mucus region compared to measurements obtained in adjacent non-consolidated regions. This combined MRE/FEM approach represents a novel way to monitor the biomechanical properties (stiffness, strain and strain gradient) that are altered in disease and therefore may lead to a novel set of diagnostic biomarkers of lung disease in pwCF as well as other patient populations.

## Methods

### Magnetic Resonance Elastography

Six normal adult volunteers n = 6 (age 24–29 yrs, 3 male, 3 female) and three pwCF (1 male and 2 females; 21–35 years old) were scanned after obtaining written informed consent. The study was approved by the Institutional Review Board (IRB) at both the Ohio State University and Nationwide Children’s Hospital. All imaging was performed on a 1.5 T MRI scanner (Avanto, Siemens Healthcare, Erlangen, Germany). All subjects were positioned supine and headfirst into the scanner. For MRE scans, mechanical vibrations at a frequency of 50 Hz were introduced separately into the right and left lungs using an in-house built acoustic driver system. The passive driver was positioned anteriorly on the apex of the lungs and the passive driver was connected to the active driver (speaker system) using a plastic tube placed outside the scan room.

### MRI and MRE Image Acquisition:

A modified spin echo-echo planar imaging (SE-EPI) sequence was used to acquire five axial slices of the lung. To minimize T2* effects and to achieve an adequate MR signal in the lungs, a SE based sequence was used. To achieve a strong signal in the lung, the shortest achievable TE of 11.6 ms was used. The sequence parameters used were: FOV: 40×40cm^2^; TR: 400ms; acquisition matrix: 128×64 interpolated to 256×256; slice thickness: 10mm; voxel size 1.56×1.56×10mm; echo train length: 9 (i.e. 7 shots to fully cover a single k-space); and 4 MRE phase offsets. For MRE, a 1:1 motion encoding gradient (MEG) was split into two unipolar lobes around the 180° refocusing pulse to achieve the minimum possible TE. Each Motion Encoding Gradient (MEG) lobe had a period of 2 ms (i.e. 250 Hz combined). In-plane and through plane mechanical motion were encoded with a 26 second breath hold for each subject at residual volume (RV).

### Lung Density Imaging:

The lung’s density (LD) changes during respiratory cycle as described previously(Holverda et al. 2011; Theilmann et al. 2009; Mariappan et al. 2011; Mariappan et al. 2014; Fakhouri, Dong, and Kolipaka 2019) and quantifying lung density is crucial for accurately estimating lung stiffness. Therefore, LD scans were performed using a fast GRE sequence(Holverda et al. 2011; Theilmann et al. 2009) with TR of 10 ms. To calculate T2* at RV, four different TEs of 1.07 (minimum TE achievable by the sequence), 1.5, 2, and 2.5ms were selected. Due to the low density of the lung at TLC relative to RV, the two shortest TEs were selected (i.e. 1.07 and 1.5ms) because of the rapid decay of the signal at TLC(Holverda et al. 2011; Theilmann et al. 2009). This scan involved a single breathhold for each, RV and TLC. A whole-body coil was used with the following parameters: FOV: 50×50cm2, slice thickness: 10mm, acquisition matrix: 64×64, and number of averages: 4.

### Calculation of Shear Stiffness

The left and right lung images were first segmented by drawing a region of interest (ROI) in each of the 5 axial slices that defined the lung outline. Next, to eliminate longitudinal and reflected waves, a 4th order Butterworth bandpass directional filter was applied in 8 directions with cutoff values of 42 − 40 waves/FOV at RV(Manduca et al. 2003). Filter cutoff values were selected based on the number of pixels manually measured in a wavelength. Third, lung shear stiffness was calculated for each slice individually by using 2D direct inversion(Manduca et al. 2001) using MRElab (Mayo Clinic, Rochester, Minnesota, USA). Finally, the resultant stiffness maps were median filtered (3×3 kernel)(Huang, Yang, and Tang 1979; Frieden 1976), and the 95th percentile(Langford 2006) of the data was used by removing outliers due to noise imbedded in the lung’s stiffness maps to report the mean stiffness value.

### Calculation of Lung Density

Lung density (LD) was estimated relative to a Gadolinium-doped water phantom that was placed on the volunteers’ chest during LD scans(Holverda et al. 2011; Theilmann et al. 2009). The MR signal received from the phantom, which is mostly water, was considered as a good representation of lung’s signal without any air. Therefore, an absolute measure of lung’s density can be estimated relative to the Gadolinium-doped water phantom. The following equation was used to obtain the initial signal (I_0_) of the lung for each pixel in the ROI,

1 $$Sm=I_{0}e^{TE*T2^{*}}$$

where Sm is the mean signal of the lungs at a given TE (i.e. 1.07, 1.5, 2, 2.5 ms). The resultant initial signal of the lung (I_0_) was then used to calculated LD in each pixel by

2 $$LD=I_{0}*CR*I_{ph}$$

where I_ph_ is the mean signal of the Gadolinium-doped water phantom, and CR is a correction factor that had to be determined due to the long decay constants of the phantom and to correct for the steady state signal that the phantom might reach at a TR of 10ms. By using the same fast GRE sequence, the CR was obtained by scanning the phantom twice with two different TRs of 10ms and 6 seconds. Then the CR was obtained by dividing mean phantom signal at TR = 6 seconds by mean phantom signal at TR = 10ms which resulted in a CR of 1.873(Holverda et al. 2011; Theilmann et al. 2009; Mariappan et al. 2014). LD was used to correct the shear modulus(Mariappan et al. 2011) and the average LD in each subject was used in the FEM models described below.

### Finite Element Modeling

**Figure 1:**
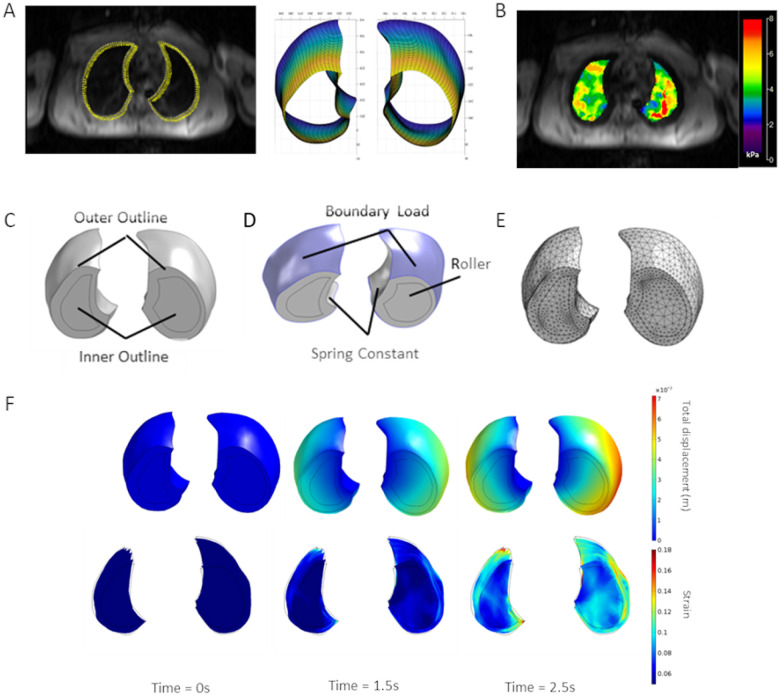
Overview of the lung finite element simulation. A) Outlines obtained in each axial MR scan using ImageJ and 3D geometry of lung section obtained by lofting outlines using NURBS toolbox. B) Example MRE stiffness map/measurements used to specify material properties in inner domain. C) Inner and outer domains in the lung section, D) Boundary conditions used in finite element simulations of breathing and E) finite element mesh used to solve governing equations. F) Example of total displacements (top row) and 1st principal strain (second row) calculated by the finite element model in a representative normal subject at end expiration (t=0), mid-respiratory cycle (t=1.5s) and end inspiration (t=2.5s).

3D FEM of lung deformation during normal tidal volume breathing was developed by inputting MRE-derived shear stiffness values at RV obtained in normal (n = 6) and cystic fibrosis (CF) (n = 3) subjects. Notably, both lungs were scanned in pwCF, and only the right lung was scanned in healthy adults. For each normal and CF lung, an outline of the lung in each axial MR scan was obtained using ImageJ and a custom written MatLab code that uses non-uniform rational basis splines (NURBS)(Spink) was used to create a 3D geometry of the lung section scanned during the MRI protocol (Fig. 1A). Due to edge-effects in the MRE-derived stiffness maps, the size of the MRE shear stiffness maps were cropped and smaller than the actual lung geometry outlines. As a result, separate inner outlines were drawn using the MRE measurements (Fig. 1B) and used to create an inner 3D geometry using NURBS. The outer and inner 3D geometries were imported into the COMSOL multi-physics finite element package and converted into a 3D solid domain (Fig. 1C). The outer geometry was used for specifying boundary conditions (Fig. 1D) while the inner geometry allowed for direct specification of spatial distributions in shear stiffness (G, referred as shear modulus in FEM). Specifically, the MRE measurements of LD corrected shear stiffness obtained in each plane (Fig. 1B) were interpolated using a 3D linear interpolation function and this function was used to directly specify shear modulus in the inner region. For these studies, the shear modulus in the outer region was assumed to be equal to the average shear modulus within the entire model. The resulting model was then meshed with quadratic tetrahedral elements (Fig. 1C) and the Solid Mechanics module in COMSOL was used to simulate lung tissue deformation during normal tidal volume respiration by specifying appropriate material models and boundary conditions.

The time-dependent equations governing tissue deformation solved in COMSOL were

3 $$\rho \frac{\partial^{2}\setminus \text{varvec}u\;}{\partial t^{2}}=\nabla •{\left(FS\right)}^{T}\;F=I+\nabla \setminus \text{varvec}u\;S=\frac{\partial W}{\partial \epsilon }\;\epsilon =\frac{1}{2}\left(F^{T}F-I\right)$$

where ρ is tissue density, **u** is the displacement field, *F* is the deformation gradient, *I* is the identity tensor, *S* is the second Piola-Kirchhoff stress tensor, *W* is the strain energy density and ε is the strain tensor. Previous studies indicated that finite element models of lung deformation that utilize a two-parameter Mooney-Rivlin hyperelastic material model yielded the highest accuracy in capturing experimentally measured motion of lung tumors (Tehrani et al. 2015). Therefore, in this study we implemented this material model by specifying

4 $$W=c_{1}\left(I_{1}-3\right)+c_{2}\left(I_{2}-3\right)+\kappa {\left(J-1\right)}^{2}\;c_{1}=c_{2}=\frac{G}{4}\;\kappa =\frac{2G(1+v)}{3(1-2v)}$$

Here, *I*_*1*_ and *I*_*2*_ are the first and second invariants of the isochoric elastic right Cauchy-Green tensor, κ is the bulk modulus and J is the elastic volume ratio. The shear modulus, G, was either specified as a constant value or specified based on MRE-derived measurements as described above. Since the lung has been modeled with a range of Poisson’s ratios, 0.2–0.5 (Zhang et al. 2004; Tehrani et al. 2015; Villard et al. 2005; Werner et al. 2009), we conducted a sensitivity analysis using the input values from Table 1 to determine the influence of Poisson’s ratio on strain and strain gradient magnitudes. Results (Supplemental Fig. 1A) indicate that strain and spatial strain gradients magnitudes are insensitive to changes in ν and therefore for all models in this study we specified ν = 0.2 since that is the most common value used in the literature.(Villard et al. 2005)

To simulate lung deformation during the respiratory cycle, several boundary conditions and loads were applied to the model (Fig. 1D). Because the patient specific geometries only represent the section of the lung scanned during the MRE protocol, a rolling/sliding boundary condition was applied to the upper and lower surfaces to restrict apical/basal movement to be in-plane only. To account for the restriction of lung deformation due to organs/tissues in the mediastinum cavity, we applied a spring foundation boundary condition to the mediastinal surfaces of the lung. Specifically, a restoring force that is linearly related to the local deformation field was applied and the spring constant in the normal and shear/tangential directions was specified as

5 $$k_{n}=\frac{E_{s}\left(1-v_{t}\right)}{d_{s}\left(1+v_{t}\right)\left(1-2v_{t}\right)}\;k_{s}=\frac{E_{s}}{d_{s}\left(2\left(1+v_{t}\right)\right)}$$

Where tissue stiffness was set to E_s_=800kPa, Poisson’s ratio to *v*_*t*_ =0.4 and tissue thickness to *d*_*s*_=10 cm. These values allowed for medial movement of the lungs without affecting the strain magnitudes within the inner region of interest. In addition, a sensitivity analysis was conducted by varying Es to investigate the effect of this parameter on simulation results, and it was determined that varying Es in the range shown in Table 1 did not affect the computed strains or strain gradients (Supplemental Fig. 1B). To simulate negative pressure ventilation, a sinusoidal vacuum pressure was applied to the outer surfaces of the lung section, and in this study, a vacuum pressure that ranged from 0 to a maximum vacuum pressure of -p was used with a sinusoidal frequency of 0.2 Hz. The maximum vacuum pressure p was determined for each patient specific model, which was based on the resultant volume ratio. Specifically, for an average normal adult, inspiration leads to an increased lung volume of ~ 0.5L (i.e. the tidal volume) about the functional residual capacity of ~ 3L. As a result, normal tidal ventilation resulted in a volume ratio change of 3.5L/3L = 1.167. As a result, p was determined in each normal adult patient specific model to give a volume ratio change of 1.167. Although the average maximum vacuum pressure in normal adults was 751.8 ± 47.3 Pa, pwCF had a 50% decreased tidal volume as compared to healthy subjects(Colasanti et al. 2004). Therefore, p was set in these models to achieve a volume ratio change of 1.083 and this resulted in an average maximum vacuum pressure in pwCF of 467.7 ± 29.0 Pa.

Simulation of lung deformation (Fig. 1F) was then used to calculate both local strain and spatial strain gradients at end inspiration in each lung. First, since we are primarily interested in tensile strain, the strain magnitude was quantified using the 1st principle strain $\epsilon_{p}^{1}$. Second, the derivatives of $\epsilon_{p}^{1}$ were used to calculate the strain gradient magnitude as

6 $$\nabla \epsilon_{p}^{1}=\sqrt{{\left(\frac{d\epsilon_{p}^{1}}{dx}\right)}^{2}+{\left(\frac{d\epsilon_{p}^{1}}{dy}\right)}^{2}+{\left(\frac{d\epsilon_{p}^{1}}{dz}\right)}^{2}}$$

### Statistical Analysis

Distributions of shear stiffness, 1st principal strain magnitude and spatial strain gradient were not found to consistently follow normal or log-normal distributions by Kolmogorov–Smirnov test. Therefore, for each subject, the median values of shear stiffness, strain magnitude and spatial strain gradient as well as the inter-quartile range (IQR) were calculated and paired t-tests or 1-way analysis of variance (ANOVA) was used to document statistical differences between median and/or IQR values. For this analysis, each lung was considered a “subject” and although both lungs were scanned in n = 3 pwCF, only the right lung was scanned in n = 6 healthy adults. Therefore, there were n = 6 “subjects”/lungs for both the healthy and CF groups. Statistical significance was determined at a p = 0.05 level.

## Results

### MRE-derived Shear Stiffness in Normal and CF Patients

**Figure 2:**
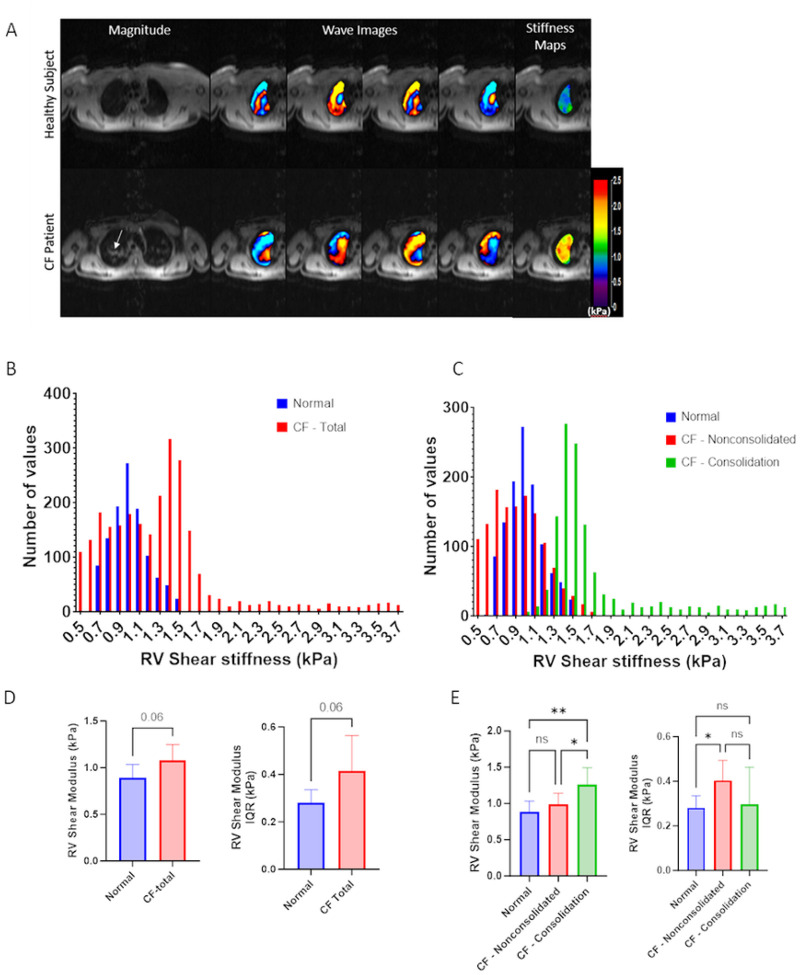
Magnetic resonance elastography data A) Wave image and shear stiffness map in representative normal and CF patients B) Shear stiffness distribution in the lung of normal and CF patients C) Shear stiffness distribution in normal lung and in consolidated and non-consolidated regions in a CF patient D) Average median shear stiffness and inter-quartile range in normal and CF subjects. E) Average median shear stiffness and inter-quartile range in the n=6 normal lungs, n=3 consolidated regions and n=6 non-consolidated regions in CF subjects.

The first objective of this study was to evaluate differences in the lung shear stiffness between healthy and CF subjects. Figure 2A shows the magnitude image and a snapshot of wave propagation images at four different time points obtained in a representative healthy adult and pwCF at RV. The shorter wavelengths (distance from red to blue region) indicated that the healthy lungs are more compliant or less stiff as compared to CF lungs. These wave propagation images were then used to calculate spatial maps of shear stiffness within all 5 axial scans (last column, Fig. 2A) and histograms of the measured shear stiffness in representative normal and CF lungs is shown in Fig. 2B. Median and interquartile range (IQR) obtained from the histograms are shown in Fig. 2D with non-significantly (p = 0.06) higher median stiffness and IQR in CF lungs. During analysis of the standard MRI magnitude images in CF lungs, there were clear regions of consolidation/mucus accumulation (see white arrow in Fig. 2A). We therefore conducted a secondary analysis where a region of interest (ROI) was defined that outlined this consolidated region on the CF lungs and this ROI was to separate the overall histograms in CF lungs into histograms of the shear stiffness both within (labeled CF-Consolidated) and outside (labeled CF-Nonconsolidated) the consolidated region (Fig. 2C). These segregated histograms were then used to calculate the median shear stiffness and IQR in the non-consolidated and consolidated (Fig. 2E). Figure 2E includes median shear stiffness and IQR in healthy lungs. Although the median shear stiffness in the non-consolidated region was not statistically different than the median shear stiffness in healthy lungs, the median shear stiffness in the consolidated region was significantly higher than both the stiffness in the non-consolidated region (p < 0.05) and the stiffness in the healthy lung (p < 0.005). Interestingly, the IQR in the non-consolidated CF lungs was statistically higher than the IQR in the healthy lungs while the IQR in the consolidated region of CF lungs was not statistically different than the IQR in either the healthy lung nor the non-consolidated region. This data clearly indicates that the consolidated region in CF lungs is stiffer than both healthy lung tissue and the surrounding non-consolidated regions. Furthermore, the large IQR values for all lungs indicates that there is significant spatial heterogeneity in shear stiffness in both the healthy and CF lungs. Although the non-consolidated regions of CF lungs were observed to have larger stiffness heterogeneity (larger IQR) than normal lungs, the consolidated regions of CF lungs have a similar heterogeneity profile (similar IQR) as normal lungs. Although these MRE measurements clearly document the heterogeneity of local lung stiffness, it is not clear how this variability in stiffness influences other dynamic mechanical parameters including strain magnitude and spatial strain gradients during tidal respiration. We therefore developed a novel computational model to address these questions.

### Heterogenous Stiffness Distributions alter Computational Predictions of Tensile Strain and Spatial Strain Gradients

Two separate computational simulations in the healthy lung models were used to determine the relative importance of heterogenous mechanical properties on tensile strain and strain gradient distributions. First, we assumed a homogenous shear stiffness value throughout the entire lung and used calculations of median shear stiffness (Fig. 2) to specify this value. Second, we developed a heterogenous computational model where the spatial variations in shear stiffness measured by MRE were directly applied in our finite element models. Prior to simulating lung deformation during tidal breathing in all healthy and CF lung models, a mesh convergence study was conducted to determine an adequate mesh density/size. For this study, a representative normal adult geometry was meshed with different size and number of quadratic elements and the outputs of 1st principal strain magnitude and spatial strain gradient were plotted as a function of the number of elements and the degrees of freedom (Supp Fig. 2). Results indicate that an average of 30,000 finite elements with 125,000 degrees of freedom yielded a sufficiently accurate solution. Therefore, all models in this study were meshed with a similar number of elements and the average number of degrees of freedom used in the adult models was 115,520 + 15,538. The mesh convergence study was conducted in a model that accounted for spatial distribution of MRE-derived stiffness.

**Figure 3:**
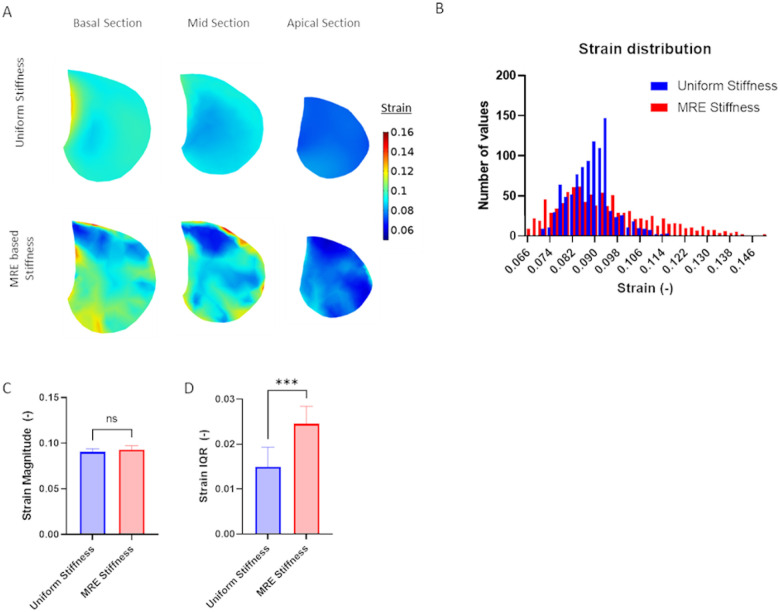
1^st^ principal strain in healthy subjects at end inspiration as calculated from computational models that either assume a homogenous stiffness (uniform) or heterogeneous stiffness based on MRE measurements. (MRE based) A) Representative maps of 1^st^ principal strain at different axial locations. B) Histograms of strain distributions in a representative subject C) Average of median strain distribution in n=6 normal adult subjects and D) average interquartile range (IQR) of strain distribution in n=6 normal adult subjects. Paired two-tailed t-test was used to document statistical significance with *** p<0.001.

Next, normal tidal respiration was simulated in each of the healthy adult models and Fig. 3A shows the spatial variations in 1st principal strain calculated in a basal, mid-lung and apical sections. All data is presented at maximum inhalation (i.e. end inspiration) and results are shown for simulations that utilize either homogenous stiffness (labeled Uniform) and heterogenous stiffness (labeled MRE-based) values. Figure 3B shows the distribution of 1st principal strains in representative homogenous and heterogenous models. Interestingly, FE models of lung deformation with homogenous stiffness exhibited a relatively narrow distribution of 1st principal strain while the MRE-based models exhibited a wider distribution of strain. The median 1st principal strain in the uniform and MRE stiffness-based lung models were not significantly different (Fig. 3B). However, the variance in strain, as measured by the strain inter-quartile range (IQR) was significantly higher in the MRE stiffness-based models as compared to the normal uniform stiffness models.

**Figure 4:**
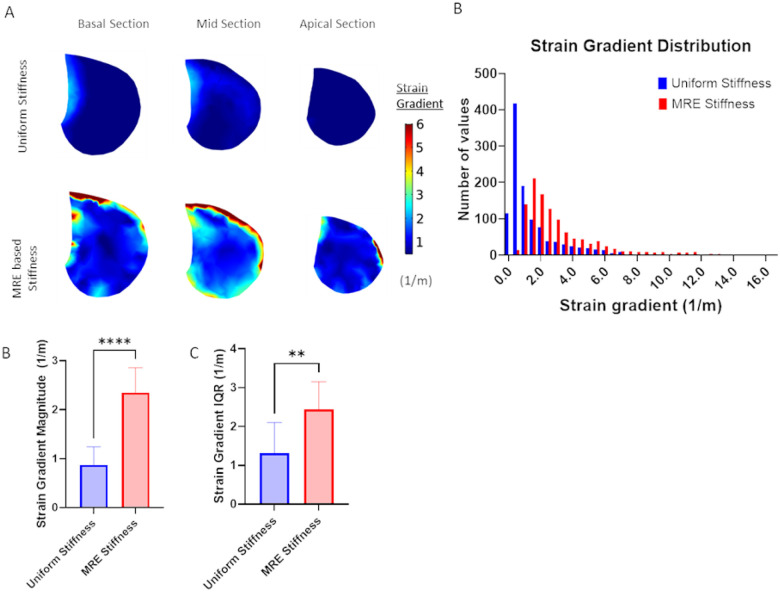
Spatial strain gradient in healthy subjects at end inspiration as calculated from computational models that either assume a homogenous stiffness (uniform) or heterogeneous stiffness based on MRE measurements. (MRE based) A) Representative maps of spatial strain gradient at different axial locations. B) Histograms of spatial strain gradient distributions in a representative subject C) Average of median spatial strain gradient distribution in n=6 normal adult subjects and D) average interquartile range (IQR) of spatial strain gradient distribution in n=6 normal adult subjects. Paired two-tailed t-test was used to document statistical significance with **p<0.01, **** p<1e-4.

As shown in Fig. 4, the spatial strain gradient was calculated in both the uniform stiffness and MRE-based stiffness models. Figure 4A shows spatial variations in the strain gradient and Fig. 4B shows representative histograms of the spatial strain gradient distributions. Models with uniform stiffness exhibited much smaller strain gradient values with a very narrow distribution while models with MRE-based stiffness exhibited a large range of significantly higher strain gradient values. The median strain gradient magnitude was significantly higher in MRE stiffness-based models (Fig. 4C) and the variance of the strain gradient magnitude as measured by the IQR was significantly higher in the MRE stiffness based models (Fig. 4D). These results clearly indicate that incorporating heterogenous stiffness properties into computational models of lung deformation during tidal respiration are required to capture the spatial heterogeneity in mechanical strain and strain gradients that might impact lung mechanobiology.

## Discussion

In this study, a novel non-invasive technique that uses MRE measurements of lung shear stiffness and FE computational analysis was used to assess regional lung stiffness (i.e. shear modulus), strain magnitude and spatial strain gradients in the lungs of pwCF compared to heathy adult lungs. Our early results indicate the degree of tissue deformation and tensile strain was highly dependent on both the spatial distribution of lung tissue stiffness and the alterations in tissue stiffness that occur in CF lung disease. An important feature of CF is mucus accumulation in the lung (Turcios 2020) that alters intrinsic biomechanical properties, as shown in Fig. 2. Alterations of these key intrinsic properties of the lung are an important contributing factor in structural changes occurring in pwCF due to chronic mucus accumulation(Esther et al. 2019). Therefore, this novel approach to assessing CF lungs has the potential for future clinical and/or research applications.

Global averages of the median shear stiffness in CF lungs did not exhibit a statistically significant higher shear stiffness compared to healthy lungs. However, when shear stiffness data in CF lungs were segregated into consolidated regions with mucus accumulation and non-consolidated regions without mucus accumulation, the averaged median shear stiffness in the consolidated regions was statistically higher than both the median stiffness in adjacent non-consolidated regions as well as in the healthy lung. The increased stiffness in the consolidated lung suggest that in regions of mucus accumulation where persistent infection occurs, scar tissue formation (Hamutcu et al. 2002) significantly alter regional tissue mechanics. These results also indicate that accounting for regional heterogeneity in mechanical properties (MRE-derived stiffness) may be an important consideration when using lung mechanics as a diagnostic parameter. For example, the initial stages of fibrosis may lead to larger regional heterogeneity in stiffness even if global measures of stiffness are unchanged.

We further investigated the importance of including heterogenous stiffness measurements in computational models by simulating respiration in two different healthy adult models that either assumed a homogenous/uniform stiffness throughout the entire lung or directly accounted for spatial distributions of MRE-derived stiffness. Although both homogenous/uniform and MRE-based models predict similar average median strain magnitude (Fig. 3C), MRE-based models, which accounted for spatial heterogeneity in stiffness, predicted significantly larger variations in median strain (Fig. 3D) and larger spatial strain gradients (Fig. 4B). Importantly, previous CT imaging studies demonstrated significant strain gradients in human lungs (Hurtado et al. 2020; Hurtado et al. 2017) and spatial gradients in strain have been shown alter fibroblast migration, orientation and contractility(Chagnon-Lessard et al. 2017; Hsieh et al. 2014; Yang et al. 2023). We, therefore, conclude that incorporating heterogeneous spatial stiffness patterns in computational simulations of lung dynamics is important since they provide a more accurate way to assess the magnitude of strain variations and spatial strain gradients and may provide additional information on disease progression and lung pathophysiology.

**Figure 5:**
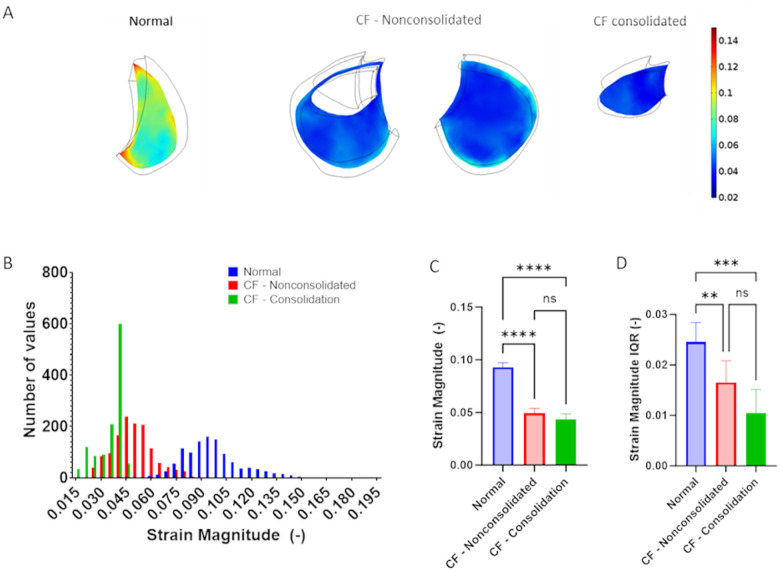
1^st^ principal strain obtained in MRE-based models of normal and CF lungs. A) Representative maps of 1st principal strain at a one axial locations in normal, CF – nonconsolidated and CF consolidated lung regions. B) Histograms of strain distributions in a representative normal and CF subject. C) Average of median of strain distribution in n=6 normal adult lungs, n=6 CF – nonconsolidated lung regions and n=3 CF consolidated lung regions and D) average interquartile range (IQR) of strain distribution in n=6 normal adult lungs, n=6 CF – nonconsolidated lung regions and n=3 CF consolidated lung regions. One-way ANOVA was used to document statistical significance with **p<0.01, *** p<0.001, and ****p<1e-4.

**Figure 6:**
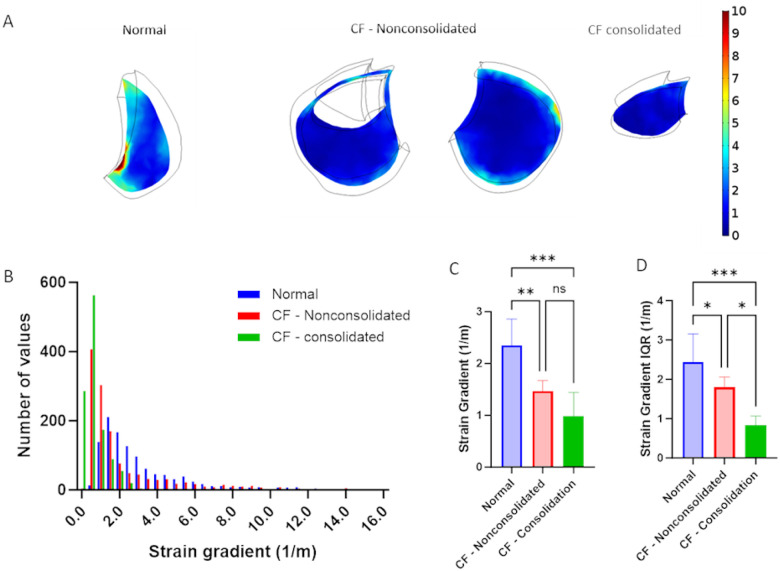
Spatial strain gradient obtained in MRE-based models of normal and CF lungs. A) Representative maps of spatial strain gradient at a one axial location in normal, CF – nonconsolidated and CF consolidated lung regions. B) Histograms of strain gradient distributions in a representative normal and CF subject. C) Average of median of strain gradient distribution in n=6 normal adult lungs, n=6 CF – nonconsolidated lung regions and n=3 CF consolidated lung regions and D) average interquartile range (IQR) of strain gradient distribution in n=6 normal adult lungs, n=6 CF – nonconsolidated lung regions and n=3 CF consolidated lung regions. One-way ANOVA was used to document statistical significance with *p<0.05, **p<0.01 and *** p<0.001.

Finally, we used MRE-FEM techniques to evaluate changes in strains and strain gradients in pwCF. Interestingly, the average median strain in both consolidated and non-consolidated lung regions was significantly less than the median strain observed in human lungs (Fig. 5BC). However, we acknowledge that this could be a result of applying lower transpulmonary pressure in the CF subjects to account for the fact that pwCF have a 50% decreased tidal volume compared to healthy subjects(Colasanti et al. 2004). Nonetheless, we found no statistically significant difference in median strain between consolidated and non-consolidated regions but did observe a statistically lower variability in strain in the consolidated regions (Fig. 5D). We also identified statistically significant lower spatial strain gradients and spatial strain gradient variability in the consolidated CF lung regions (Figs. 6C and 6D). Together, this data indicates that mucus accumulation in the CF lung drastically alters the local mechanical environment and primarily leads to a decrease in strain/deformation gradients as opposed to changes in overall strain/deformation magnitude. Therefore, assessing strain variability and/or spatial strain gradients via our MRE-FEM method may represent an important new way to track clinically relevant changes in lung micromechanics and therefore a better way to track disease progression.

Although our MRE-FEM technique represents an innovative way to non-invasively assess the heterogenous mechanical properties of the lung, we acknowledge that several improvements are needed in the future. First, the current MR scanning protocol requires patients to hold their breath for ~ 26 seconds. However, our group has recently developed innovative free-breathing sequences (Fakhouri et al. 2022) and future studies could use these sequences to obtain MRE-derived shear stiffness measurements. Second, in this study, only 5 axial scans were obtained and thus only a portion of the lung was modeled using FEM. Future studies need to obtain a larger number of axial slices that cover a larger portion of the lung. Finally, the lung is a poro-viscoelastic tissue and future FEM simulations could use recently describe techniques to account for these properties(Li et al. 2021).

In summary, we have developed a novel technique using MRE and FEM to non-invasively assess regional lung stiffness (i.e. shear modulus), strain magnitude and spatial strain gradients in pwCF compared to healthy controls. Results indicate that integrating heterogeneous MRE stiffness measurements into FEM of lung deformation during respiration yields significantly more variability in strain and larger strain gradients consistent with CT-based observations. Results also indicate that accurate analysis of MRE stiffness and FEM strains/strain gradients in pwCF requires segregation of the data into consolidated and non-consolidated regions. Specifically, the MRE-FEM technique documented differences in strain variability and spatial strain gradients between consolidated and adjacent non-consolidated regions. Therefore, it can be concluded that heterogenous stiffness distributions in both the CF and healthy lungs should be accounted for in computational models that seek to characterize the local mechanical environment in the lung. Furthermore, this study highlights the limitation of current *in-vitro* systems which typically utilize uniform deformation fields to study the mechanobiology of lung cells and highlights the need to develop novel systems that can investigate the role of spatial strain gradient in the mechanobiological mechanisms of pulmonary disease.

## Figures and Tables

**Table 1.** Biomechanical parameter values used for sensitivity analysis.

Parameter	Description	Value Range	Baseline Parameter Value
v	Poisson’s Ratio	0.2 to 0.5	0.2
Es	Spring Stiffness	800 to 8e10 Pa	8 kPa
Go	Outer Lung Shear Stiffness	500 to 10000 Pa	3000 Pa
P	Boundary Load	100 to 1000 Pa	1000 Pa

## Abbreviations

MRE: magnetic resonance elastography
pwCF: people with Cystic Fibrosis
FEM: finite element models
COPD: chronic obstructive pulmonary disease
PC: phase-contract
MRI: magnetic resonance imaging
TLC: total lung capacity
RV: compared to residual volume
CT: Computed Tomography
SE-EPI: spin echo-echo planar imaging
FOV: field of view
LD: Lung density
